# Ibogaine Detoxification Transitions Opioid and Cocaine Abusers Between Dependence and Abstinence: Clinical Observations and Treatment Outcomes

**DOI:** 10.3389/fphar.2018.00529

**Published:** 2018-06-05

**Authors:** Deborah C. Mash, Linda Duque, Bryan Page, Kathleen Allen-Ferdinand

**Affiliations:** ^1^Department of Neurology, Leonard M. Miller School of Medicine, Miami, FL, United States; ^2^Department of Molecular and Cellular Pharmacology, Leonard M. Miller School of Medicine, Miami, FL, United States; ^3^Department of Anthropology, University of Miami, Coral Gables, FL, United States; ^4^General Medical Practice, Basseterre, Saint Kitts and Nevis

**Keywords:** noribogaine, ibogaine, detoxification, withdrawal, craving, opioid dependence

## Abstract

Ibogaine may be effective for transitioning opioid and cocaine dependent individuals to sobriety. American and European self-help groups provided public testimonials that ibogaine alleviated drug craving and opioid withdrawal symptoms after only a single dose administration. Preclinical studies in animal models of addiction have provided proof-of-concept evidence in support of these claims. However, the purported therapeutic benefits of ibogaine are based on anecdotal reports from a small series of case reports that used retrospective recruitment procedures. We reviewed clinical results from an open label case series (*N* = 191) of human volunteers seeking to detoxify from opioids or cocaine with medical monitoring during inpatient treatment. Whole blood was assayed to obtain pharmacokinetic measures to determine the metabolism and clearance of ibogaine. Clinical safety data and adverse events (AEs) were studied in male and female subjects. There were no significant adverse events following administration of ibogaine in a dose range that was shown to be effective for blocking opioid withdrawal symptoms in this study. We used multi-dimensional craving questionnaires during inpatient detoxification to test if ibogaine was effective in diminishing heroin and cocaine cravings. Participants also completed standardized questionnaires about their health and mood before and after ibogaine treatment, and at program discharge. One-month follow-up data were reviewed where available to determine if ibogaine’s effects on drug craving would persist outside of an inpatient setting. We report here that ibogaine therapy administered in a safe dose range diminishes opioid withdrawal symptoms and reduces drug cravings. Pharmacological treatments for opioid dependence include detoxification, narcotic antagonists and long-term opioid maintenance therapy. Our results support product development of single oral dose administration of ibogaine for the treatment of opioid withdrawal during medically supervised detoxification to transition drug dependent individuals to abstinence.

## Introduction

Ibogaine is an indole alkaloid isolated from the roots of the West African shrub Tabernanthe iboga. The therapeutic and oneirophrenic (dream-like) effects of iboga roots have been described in the ethnobotanical literature for centuries, where ingestion of Ibogaine root preparations ceremonial and medicinal use (Goutarel et al., 1993; Samorini, 1995). In Africa, approximately 2–3 million members of the Bwiti religion in Gabon, Zaire, and the Cameroun take large doses for “the ‘Bwiti initiation ritual’ – a powerful ‘rebirth’ ceremony that group members typically undergo before the commencement of their teenage years” (Fernandez and Fernandez, 2001).

Ibogaine was marketed in France in the pharmaceutical preparation Lambarene (200 mg tablet). The root extract contained approximately 5 mg ibogaine and other minor iboga alkaloids. In the early 20th century, this preparation was marketed as a neuromuscular stimulant at a dose of 2–4 tablets/day (Goutarel et al., 1993). Several groups reported on the potential benefit of ibogaine for the treatment of drug dependence (Lotsof, 1985; Sheppard, 1994; Luciano, 1998; Mash et al., 1998; Alper et al., 1999). Academic researchers reported descriptions of robust effects of the drug in preclinical animal models and *in vitro* data were obtained which identified possible mechanism(s) of action (Glick et al., 1994, 2000; Popik et al., 1995; Mash et al., 1995; Staley et al., 1996; Baumann et al., 2001; for review, Belgers et al., 2016; Mash et al., 2016).

Despite the fact that ibogaine was never approved as a medicine for the treatment of drug addiction in most western countries (Vastag, 2005; IND39,680), human experience suggested the effectiveness of single large doses of ibogaine to block withdrawal symptoms and cravings in drug dependent individuals (Sheppard, 1994; Alper et al., 1999; Mash et al., 2000, 2001; Lotsof and Alexander, 2001; Bastiaans, 2004). Self-treating heroin addicts made the original discovery in the 1960s that ibogaine eliminates the signs and symptoms of opioid withdrawal. Alper and coworkers collected data from people who took ibogaine between 1962 and 1993 with the intention of “interrupting” their heroin addiction (Alper et al., 1999). Out of 33 human subjects treated with 6–29 mg/kg ibogaine (average 19 mg/kg), 25 reported blockade of opioid withdrawal symptoms and no further desire to take heroin in the days following treatment. We reported results for a small case series following lower oral doses of ibogaine (10–12 mg/kg) in patients who had undergone pre-treatment screening and physical evaluation (Mash et al., 2000, 2001). Objective physician ratings demonstrated that ibogaine reduced opiate withdrawal scores in twenty seven heroin dependent patients. Patients reported diminished opioid craving and significantly improved mood after treatment. Interestingly, these effects appeared to persist over a long period of time based on self-reports at a 1-month follow up interview. The recent observational studies from Mexico (Brown and Alper, 2017; Davis et al., 2017) and New Zealand (Noller et al., 2018) endorse the efficacy of ibogaine as pharmacological treatment for opioid detoxification (Mash, 2018).

Although 1000s of patients suffering from opioid use disorder have been treated with ibogaine, clinical efficacy data from the published case series are hardly comparable, and the reports vary widely with regard to the assessment of outcome measures. Also, there have yet to be any clinical trials to demonstrate efficacy of the drug for opioid dependence. Like most CNS drugs, ibogaine is a highly lipophilic compound that is subject to complex biotransformation and variable half-life due to genetic polymorphisms (Obach et al., 1998; Mash et al., 1998, 2000, 2001). This issue among other lingering concerns for patient safety continue to hinder the drug development of ibogaine in the US or elsewhere.

Heroin and prescription opioid dependence is a growing concern that has great societal impact and rising health care costs in the hundreds of billions of dollars (Cornish et al., 2010; Degenhardt et al., 2011; Volkow et al., 2014; Kolodny et al., 2015). We report here clinical observations of ibogaine treatments taken from an open label study to assess the safety and efficacy of ibogaine in individuals seeking detoxification from opioids and cocaine. The results demonstrate that medically assisted ibogaine detoxification affords a safe and effective method to discontinue substance dependence and misuse.

## Materials and Methods

### Inclusion and Exclusion Criteria

Individuals participated in a 12-day inpatient study to determine the safety and open-label efficacy of ibogaine as a pharmacological treatment for managing withdrawal symptoms. The study was conducted in a 12-bed freestanding facility in St. Kitts, West Indies. The treatment program had a planned duration of 12 days and stated goals of: (1) safe physical detoxification from opioids or cocaine, (2) motivational counseling, and (3) referral to aftercare programs and community support groups (12-step programs) (Mash et al., 1998, 2000). Subjects were self-referred for inpatient detoxification and met inclusion/exclusion criteria. All participants signed an informed consent at program entry to allow medical record review of study results for submission to the Food and Drug Administration (FDA). Retrospective chart review of patient records was conducted under University of Miami Institutional Review Board approval (IRB 04-0366). All individuals were subjected to a physician’s review of the history and physical examination, clinical laboratory results and electrocardiograms for inclusion in the study. The results of the electrocardiogram and clinical laboratory testing were within predetermined normal limits at program entry. Exclusion criteria included histories of stroke, epilepsy and axis I psychotic disorders, cardiovascular and liver pathology, and HIV/AIDS.

### Oral Dose Ibogaine

Participants included 191 self-referred treatment seeking opioid and cocaine dependent men (*n* = 144) and women (*n* = 47). All subjects met DSM-IV criteria for opioid or cocaine dependence and demonstrated active use with positive urine screens at program entry to the study. Participants were administered oral doses of ibogaine HCl (8–12 mg/kg) in gel caps under open-label conditions. Opioid dependent patients were switched at program entry to morphine sulfate (Oral Morphine Solution 10 mg/5 ml) for opioid withdrawal control prior to ibogaine detoxification. Safety evaluations included physical examinations, physician ratings of AEs, safety laboratory tests, vital signs, 12-lead ECG and ECG telemetry from -1 h to 24 h. Whole blood concentrations of ibogaine and noribogaine were measured using a validated GC/MS method with deuterated internal standards (Hearn et al., 1995). Ibogaine and noribogaine concentrations above the lower limit of quantification were used to calculate pharmacokinetic parameters. Subjects were genotyped for the CYP2D6 alleles as described previously (Heim and Meyer, 1992).

On admission, participants were administered the Addiction Severity Index (McLellan et al., 1992, 1999) and participated in the Structured Clinical Interview for DSM-IV Axis I Disorders (SCID I). Trained psychiatrists administered this interview. In cases where the participant’s responses were deemed questionable due to intoxication or withdrawal signs, portions of all interviews were re-conducted later as necessary. A comprehensive psychosocial assessment was used to obtain additional information about substance use history, and past and current medical condition(s) later cross-referenced to ensure accuracy. Physicians assessed opioid withdrawal signs and symptoms on the day of treatment, before and after ibogaine administration (OOWS, range 0–13; Handelsman et al., 1987).

Participants were required to complete a series of standardized self-report instruments to assess drug craving and mood at different time points during the study and, if available to our research staff, at 1 month following discharge. Subjects were asked to provide ratings of their current level of craving for cocaine or opioids using questions from the Heroin (HCQ-29) and Cocaine (CCQ-45) Craving Questionnaires (Tiffany et al., 1993; Singelton, 1998; Heinz et al., 2006). The HCQ is designed to capture five theoretically distinct conceptualizations of drug craving: (1) desire to use, (2) intention to use, (3) anticipation of positive outcome, (4) anticipation of relief from withdrawal or dysphoria, and (5) lack of control over use.

### Mood and Craving Measures

The depressive symptoms were measured using the Beck Depression Inventory version II (BDI-II) (Beck et al., 1996a,b), Profile of Moods (POMS, 2nd edition) and Symptoms Checklist-90 scales (SCL-90). Subjects’ scores from the Beck Depression Inventory, POMS, SCL-90 and the HCQ-29 and CCQ-45 craving subscales were analyzed by primary drug of abuse (opioids or cocaine) across treatment phase (pre-ibogaine, post-ibogaine, and 30 days after discharge). A repeated measures mixed model analysis of variance with time post-treatment on Days 5 (discharge) and Day 30 (1 month follow up) was performed with days as a repeated measure on subject with the change from baseline score as the dependent variable. A compound symmetric covariance model was used to control for the correlation between the two repeated measures (SAS Software, SAS Institute, Cary, NC, United States). Patients with incomplete data were included in the analyses, after assessment of the data to determine whether missing data were informative or not. Missing data by excluding patients with incomplete data or imputing data was examined and ruled out as potentially producing biased results by comparing the mean change from baseline for post treatment discharge and 30 day follow up assessments. For all analyses, the criterion for significance was *p* ≤ 0.05, two-tailed.

### Elicitation Narratives

A licensed therapist worked with the subjects to provide psychological support during and after administration of ibogaine. A semi-structured elicitation narrative was used to capture perceptual changes and subjects’ interpretation of the drug effects. Subjects narrated their subjective experience to oral doses of ibogaine HCL within 3 days after receiving an oral dose of ibogaine. The interviewer, trained in open-ended elicitation techniques, obtained descriptions of the acute drug effects and perceived benefits of ibogaine by use of an initial stimulus question and a guided questionnaire format. The tape recordings of elicited interviews were transcribed verbatim onto word processing files. Once transcribed, we used a content coding system based on a modified version of the Outline of Cultural Materials (Murdock et al., 1987) to mark places in each of the interviews where key elements of content appeared. We repeated the analysis and cross-coding of the transcription until the coders achieved greater than 90% agreement.

## Results

### Demographics of Opioid and Cocaine Dependent Subjects

The demographic characteristics of the opioid and cocaine dependent subjects are shown **Tables 1, 2**. The opioid and cocaine subjects did not differ significantly in the majority of the socio-demographic and clinical measures of addiction severity investigated at the baseline (Supplementary Tables S1, S2). The average age of the opioid abusers was 35.8 ± 9.9 years. The subjects were habitual users with at least 5.5 ± 7.2 prior treatment admissions for their opioid dependence and 19.2 ± 13.0 days of use in the past month before treatment. Lifetime use was 11.2 ± 8.6 years. Most of the subjects were Caucasian (95.1%) and male (67%) in this study. There was a high rate of depressive disorders reported with 52.9% meeting clinical criteria for major depressive disorder or depression NOS in agreement with prior reports (Grant, 1995).

**Table 1:** Demographic characteristics of opioid dependent subjects.

Variable	Total (*N* = 102)	Female (*n* = 34)	Male (*n* = 68)
Age, Mean ± SD	35.8 ± 9.9	33.0 ± 9.1	37.1 ± 10.1
Ethnicity % of subjects			
Caucasian	95.1% (*n* = 97)	33.3% (*n* = 34)	61.8% (*n* = 63)
African American	0%	0%	0%
Hispanic	2.9% (*n* = 3)	0%	2.9% (*n* = 3)
Native American	2.0% (*n* = 2)	0%	2.0% (*n* = 2)
Years of Education, Mean ± SD	15.2 ± 3.2	14.8 ± 2.5	15.3 ± 3.4
Years of Opioid Use, Mean ± SD	11.2 ± 8.6	8.9 ± 6.8	12.4 ± 9.3
Days of Opioid Use in Past 30 prior to treatment, Mean ± SD	19.2 ± 13.0 (*n* = 95)	19.1 ± 13.1 (*n* = 31)	19.3 ± 13.0 (*n* = 64)
Previous Drug Treatments, Mean ± SD	5.5 ± 7.2	5.5 ± 5.5	5.5 ± 8.0
*Coexisting Axis I – II Disorders*			
Anxiety Disorders except PTSD	20.6% (*n* = 21)	23.5% (*n* = 8)	19.1% (*n* = 13)
Bipolar Disorder	3.9% (*n* = 4)	0%	5.9% (*n* = 4)
Depressive Disorders	52.9% (*n* = 54)	64.7% (*n* = 22)	47.1% (*n* = 32)
Obsessive-Compulsive Disorder	4.9% (*n* = 5)	5.9% (*n* = 2)	4.4% (*n* = 3)
Posttraumatic Stress Disorder	8.8% (*n* = 9)	17.7% (*n* = 6)	4.4% (*n* = 3)
Attention-Deficit Disorder (II)	5.9% (*n* = 6)	2.9% (*n* = 1)	7.4% (*n* = 5)
Antisocial Personality Disorder (II)	19.6% (*n* = 20)	20.6% (*n* = 7)	19.1% (*n* = 13)
Borderline Personality Disorder (II)	18.6% (*n* = 19)	32.4% (*n* = 11)	11.8% (*n* = 8)
Schizotypal/Schizophreniform PD (II)	19.6% (*n* = 20)	11.8% (*n* = 4)	23.5% (*n* = 16)

**Table 2:** Demographic characteristics of cocaine dependent subjects.

Variable	Total (*N* = 89)	Female (*n* = 13)	Male (*n* = 76)
Age, Mean ± SD	36.1 ± 9.1	35.1 ± 5.9	36.3 ± 9.6
Ethnicity, % of subjects			
Caucasian	78.7% (*n* = 70)	13.5% (*n* = 12)	65.2% (*n* = 58)
African American	2.2% (*n* = 2)	0%	2.2% (*n* = 2)
Hispanic	15.7% (*n* = 14)	0%	15.7% (*n* = 14)
Native American	3.4% (*n* = 3)	1.1% (*n* = 1)	3.4% (*n* = 2)
Years of Education, Mean ± SD	14.1 ± 2.2	14.6 ± 2.1	14.0 ± 2.2
Years of Cocaine Use, Mean ± SD	13.1 ± 6.4	14.0 ± 8.1	13.0 ± 6.1
Days of Cocaine Use in Past 30 prior to treatment, Mean ± SD	9.1 ± 10.7 (*n* = 82)	15.3 ± 14.2 (*n* = 12)	8.2 ± 9.8 (*n* = 69)
Number of Previous Drug Treatments, Mean ± SD	5.1 ± 6.1	9.1 ± 8.9	4.4 ± 5.2
*Coexisting Axis I – II Disorders*			
Anxiety Disorders except PTSD	11.2% (*n* = 10)	7.7% (*n* = 1)	11.8% (*n* = 9)
Bipolar Disorder	23.6% (*n* = 21)	23.1% (*n* = 3)	23.7% (*n* = 18)
Depressive Disorders	40.4% (*n* = 36)	46.2% (*n* = 6)	39.5% (*n* = 30)
Obsessive-Compulsive Disorder	1.1% (*n* = 1)	0%	1.3% (*n* = 1)
Posttraumatic Stress Disorder	4.5% (*n* = 4)	0%	5.3% (*n* = 4)
Attention-Deficit Disorder (II)	22.5% (*n* = 20)	7.7% (*n* = 1)	25.0% (*n* = 19)
Antisocial Personality Disorder (II)	27.0% (*n* = 24)	0%	31.6% (*n* = 24)
Borderline Personality Disorder (II)	28.1% (*n* = 25)	23.1% (*n* = 3)	29.0% (*n* = 22)
Schizotypal/Schizophreniform PD (II)	7.9% (*n* = 7)	7.7% (*n* = 1)	7.9% (*n* = 6)

The cocaine dependent subjects had an average age of 36.1 ± 9.1 years and 13.1 ± 6.4 years of lifetime use at program admission. A total of 78.7% were Caucasian and 85% were male. The number of previous substance abuse treatments was 5.1 ± 3.1, confirming a high rate of relapse for cocaine abusers seeking treatment with ibogaine. The rate of depressive disorders for cocaine dependent subjects was 40.4%, slightly lower than for subjects meeting criteria for opioid use disorder. In contrast, we observed a higher rate of comorbidity for bipolar disorder (23.6%) in the cocaine abusers compared to the opioid dependent subjects. Attention deficit disorder was also higher in cocaine abusers (22.5%) compared to opioid dependent subjects (5.9%).

Thus, this observational case series included mostly male opioid (67%) and cocaine (85%) dependent subjects that were admitted for ibogaine treatment. Based on the limitation of the sample size and the open label study design, we did not attempt to test for gender differences in safety, pharmacokinetics or efficacy measures.

### Safety and Cardiovascular Changes in Vital Signs

Ibogaine was well tolerated in this study in male and female subjects, with nausea and vomiting and ataxia of gait as the most common side effects observed shortly after drug administration. There were no changes noted on physical examination or safety laboratory tests across the dose range administered. Most subjects commonly reported perceptual changes during the drug absorption phase, which subsided usually within 4–6 h after ibogaine administration. Headache was a common complaint reported post dose in 7% of the subjects. Rebound headaches after discontinuation of opioid analgesic use are common complaints due to chronic misuse, which may explain in part the incidence observed in this study, since all but one complaint of headache post ibogaine was reported by an opioid dependent subject (data not shown). There were no serious AEs that occurred in this study. Orthostatic hypotension occurred in 5% of the subjects. Of these AEs, approximately 2% were judged to be of moderate severity. We observed several cases of orthostatic hypotension and bradycardic heart rate early after ibogaine administration in cocaine dependent subjects. Interestingly, this effect of ibogaine was not observed in our study in opioid abusers (data not shown). Volume depletion (either acute or subacute) that was a likely consequence of cocaine abuse was recognized by our consulting cardiologist as a likely cause of orthostatic hypotension, since administration of intravenous fluids rapidly normalized symptomatic bradycardia and hypotension. Because of this observation (Mash et al., 2000), we routinely administered intravenous fluids 1 h prior to ibogaine administration to all patients to ensure safety regardless of their drug dependency disorder.

There were no ocular or visual side effects noted in this study at post-dose physician examinations and none of the patients had any complaints of pronounced dry eye, ocular pain, eye redness or eye discomfort. Clinical laboratory test results were in the normal range for white blood cell count, neurotrophic levels, sodium and potassium levels. Liver function (ALT, AST, ALP and GGT) was unchanged from baseline measures following ibogaine administration.

### Ibogaine Pharmacokinetics and Opioid Withdrawal

**Table 3** illustrates representative data comparing pharma-cokinetics of ibogaine and noribogaine, CYP2D6 genotypes and OOWS ratings (*N* = 22). Opioid withdrawal symptoms were recorded before ibogaine administration, approximately 12 h after the last dose of oral morphine. Withdrawal symptoms ranged between 3 and 13 (0–13 maximal score) for these subjects. The lower pre-dose OOWS ratings were seen in subjects withdrawing from methadone, which likely accounts for the lower number of acute withdrawal signs and symptoms seen for some of the opioid dependent subjects that were switched from methadone to oral morphine (data not shown).

**Table 3:** Comparison of pharmacokinetic data and opioid withdrawal ratings by genotype.

	Ibogaine						Noribogaine					
	Dose mg	Code	*T*_max_ (h)	*C*_max_ ng/ml	t1/2 (h)	AUCinf mg/h/kg	*T*_max_ (h)	*C*_max_ ng/ml		CYP 2D6	OOWS Pre-dose	OOWS Post-dose
1	500	F12	1.5	900	6.1	7.1	8	397		wt/4	5	0
2	500	F10	2	1075	3.9	5.7	6	518		wt/4	12	2
3	700	M6	4	940	2.8	8.46	2	452		wt/4	8	0
4	800	M26	1	468	1.8	2.09	6	763		wt/4	5	0
5	800	M13	2	1245	4.3	10.4	22	673		wt/4	5	1
6	800	M11	1	1753	3.2	10.6	8	632		wt/4	5	0
7	800	M21	4	1300	4.7	15.7	22	217		wt/4	5	0
8	500	M5	NQ	NQ	1.3	0.98	4	1339		wt/wt	7	2
9	500	F20	2	531	1.7	3.36	8	1294		wt/wt	5	0
10	500	F2	1.5	39	NQ	0.82	6	706		wt/wt	5	0
11	600	M14	1	464	2.4	2.94	4	624		wt/wt	8	0
13	750	M30	1	1178	1.8	1.46	8	874		wt/wt	3	2
14	750	F31	4	885	NQ	5.75	8	1033		wt/wt	12	2
16	800	M16	0.5	1425	1.1	2.67	4	1164		wt/wt	3	1
17	800	M41	2	1330	3.8	7.65	6	1606		wt/wt	10	1
18	800	M63	2	653	3.8	3.43	4	1250		wt/wt	6	1
19	800	M18	1.5	823	2.7	4.08	4	962		wt/wt	5	2
20	800	M53	4	986	3.6	10	4	1027		wt/wt	13	1
21	900	M62	4	1122	2.8	8.35	12	1176		wt/wt	5	1
22	1000	M15	1.3	1251	2.2	6.47	6	1194		wt/wt	13	1

*Representative pharmacokinetic measures of ibogaine and noribogaine in whole blood samples from opioid dependent subjects seeking detoxification. CYP2D6 genotypes (wt/4) intermediate and (wt/wt) fast metabolizers. OOWS, Objective Opiate Withdrawal Rating Scale, Physician-rated, 13-item; total score pre-ibogaine administration and post-dose (~ 36 h after last dose of morphine).*

**FIGURE 1:**
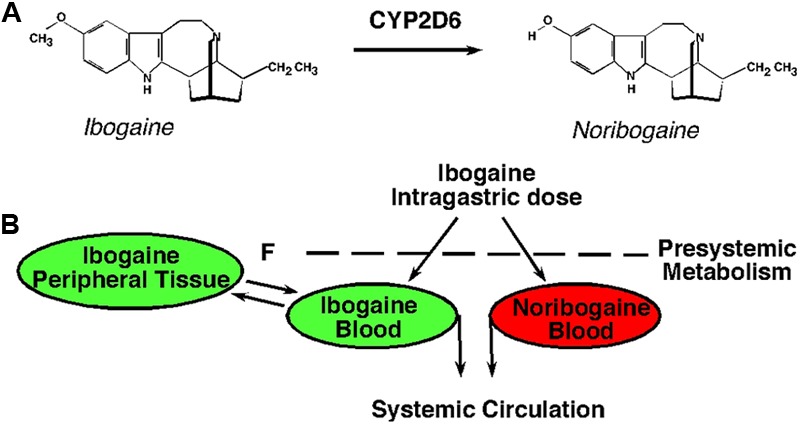
Ibogaine metabolism to noribogaine. **(A)** Molecular structures of ibogaine and noribogaine illustrate that ibogaine undergoes *O*-demethylation to form 12-hydroxyibogamine (noribogaine) by the action of cytochrome P4502D6 (CYP2D6). **(B)** Ibogaine is metabolized to noribogaine in the gut wall and liver. Genetic polymorphisms of CYP2D6 influence the biotransformation of ibogaine in humans, resulting in complex pharmacokinetics (fast, slow, and intermediate metabolizers).

Ibogaine is metabolized in the gut wall and liver by the action of cytochrome P4502D6 (**Figure 1**; Obach et al., 1998). Ibogaine is rapidly converted to 12-hydroxyibogamine (noribogaine) with a *T*_max_ observed between 0.5 and 4 h. The time to reach a *T*_max_ for noribogaine was consistent with first pass metabolism post dose administration of ibogaine (**Figure 1**). *C*_max_ values (ng/ml) are shown for ibogaine and noribogaine in opioid dependent patients by genotype. Subjects 8 (M5, male) and 10 (F2, female) were ultra-rapid metabolizers, resulting in blood levels below the level of assay detection for ibogaine. Both subjects reported an absence of any oneirophrenic (dream-like) properties of ibogaine, consistent with the low levels of ibogaine measured in blood (Mash et al., 1998, 2001). Physician rated withdrawal signs and symptoms at post dose assessments were markedly reduced compared to pre-dose baseline withdrawal severity measures. Objective signs of opioid withdrawal were mild and none were exacerbated at later time points, in keeping with the extended half-life of noribogaine (**Table 3**).

### Drug Craving and Mood Following Ibogaine Detoxification

Ibogaine subjects undergoing opioid detoxification reported significantly decreased drug craving on five measures taken from the heroin (HCQ-29) craving questionnaire post-treatment and 1 month follow up assessments compared to baseline measures (**Table 4**; *p* < 0.0001). The HCQ-29 uses questions that investigate about specific aspects of drug craving, including urges and plans to use drug, the positive reinforcing effects of the drug, and the expectation of positive benefits from using heroin or cocaine to alleviate withdrawal. A common feature is the lack of self-control over drug use which is most operative under conditions of active use and in subjects at high risk for relapse.

**Table 4:** Self-reported dimensions of craving of opioid dependent participants.

Subscale	Pre-Ibogaine (*N* = 75)	Discharge (*N* = 74)	1 Month (*N* = 37)	*F*	*P*
HCQ-NOW Factor 1: Emotionality (Negative mood state)	3.51 (0.22)	2.02 (0.14)	1.69 (0.19)	26.53	0.0001
HCQ-NOW Factor 2: Purposefulness (Desire or intent to use drug now)	4.10 (0.23)	2.21 (0.15)	2.04 (0.22)	33.36	0.0001
HCQ-NOW Factor 3: Compulsivity (Lack of confidence in ability to quit using drug)	3.23 (0.19)	2.04 (0.13)	1.64 (0.14)	23.62	0.0001
HCQ-NOW Factor 4: Expectancy (Expected positive benefits of drug use)	4.51 (0.20)	3.74 (0.19)	2.90 (0.29)	11.47	0.0001

*Incomplete or item-level missing data on one or more dimensions of craving or rating scale resulted in the exclusion of the questionnaires from the computation of total score means.*

Opioid dependent patients had significant reductions in the mean scores for all five of these domains of craving measured at program discharge. Cocaine craving questionnaires (CCQ-29) demonstrated ibogaine detoxification was effective in blocking drug craving (**Table 5**; *p* < 0.0001). Similar results were obtained using the Minnesota Cocaine Craving Scale (Halikas et al., 1991). We observed that the three-factor assessment of cocaine craving (intensity, frequency and duration) demonstrated significant reductions in the severity of self-reported cocaine craving measures at program discharge (**Table 5**; *p* < 0.0001). In person follow-up assessments were obtained, 1 month after program discharge whenever possible. The results demonstrated beneficial after effects of ibogaine detoxification on drug cravings in opioid and cocaine dependent subjects reported at 1-month assessments. The results demonstrate that opioid and cocaine craving scores were significantly decreased across all subscales (Factors 1–4; **Tables 4, 5**). The main effect of time after detoxification was significant (*p* < 0.001). The MCCS scores of craving intensity, frequency and duration were significantly lower in the cocaine group (*p*-values ≤ 0.05).

**Table 5:** Self-reported dimensions of craving of cocaine dependent participants.

Subscale	Pre-Ibogaine (*N* = 81)	Discharge (*N* = 79)	1 Month (*N* = 32)	*F*	*p*
CCQ-NOW Factor 1: Emotionality (Negative mood state)	1.85 (0.13)	1.09 (0.03)	1.19 (0.05)	22.11	0.0001
CCQ-NOW Factor 2: Purposefulness (Desire or intent to use drug now)	2.60 (0.14)	1.54 (0.20)	1.57 (0.09)	28.37	0.0001
CCQ-NOW Factor 3: Compulsivity (Lack of confidence in ability to quit using drug)	4.27 (0.16)	2.95 (0.13)	3.15 (0.20)	24.44	0.0001
CCQ-NOW Factor 4: Expectancy (Expected positive benefits of drug use)	2.51 (0.14)	1.93 (0.11)	1.76 (0.20)	8.60	0.0003
Minnesota Cocaine Craving Scale (MCCS)	Pre-Ibogaine	Discharge	1 Month	*F*	*p*
MCCS Factor 1: Craving Intensity	5.51 (0.38) (*n* = 83)	1.47 (0.14) (*n* = 74)	1.96 (0.23) (*n* = 25)	56.35	0.0001
MCCS Factor 2: Craving Frequency	2.28 (0.19) (*n* = 83)	0.29 (0.10) (*n* = 75)	0.52 (0.51) (*n* = 25)	46.42	0.0001
MCCS Factor 3: Craving Duration	2.51 (0.24) (*n* = 81)	1.36 (0.14) (*n* = 73)	1.21 (0.12) (*n* = 24)	10.75	0.0001

*Incomplete or item-level missing data on one or more dimensions of craving or rating scale resulted in the exclusion of the questionnaires from the computation of total score means.*

Depression severity was determined before and after ibogaine by the Beck Depression Inventory (BDI) and compared to self-reported scores on the POMS and SCL-90-R where available. Self–reported measures of depression symptoms are shown for opioid and cocaine dependent subjects (**Tables 6, 7**). The Beck Depression Inventory total score means were significantly decreased at 1-month follow up assessments compared to pre-ibogaine baseline and program discharge (*p* < 0.001). The BDI has been validated as a screening and diagnostic instrument for depression using a cut off value of 13/14 (Furlanetto et al., 2005). Baseline BDI total score mean was 16.53 (*N* = 88), in keeping with the high percentage of depressive symptoms diagnosed on SCID-I clinical assessments. Ibogaine detoxification was associated with a rapid improvement in mood scores for opioid dependent subjects across all three assessments (BDI, POMS and SCL-90-R; **Table 6**). These results demonstrate that ibogaine reduced withdrawal severity and depressive symptoms following abrupt cessation of opioid use.

**Table 6:** Self-reported depressive symptoms in opioid dependent subjects.

	Pre-Ibogaine	Discharge	1 Month	*F*	*P*
Beck Depression Inventory Total Score Mean	16.5 (3.8) (*n* = 88)	8.9 (2.1) (*n* = 82)	4.5 (1.9) (*n* = 32)	29.79	0.0001
POMS Depression Subscale Total Depression/Dejection Mean	22.1 (14.7) (*n* = 85)	10.8 (11.2) (*n* = 87)	5.8 (7.3) (*n* = 30)	24.45	0.01
SCL-90-R Depression Subscale Total Depression Mean	1.7 (0.9) (*n* = 85)	0.8 (0.7) (*n* = 86)	0.4 (0.6) (*n* = 28)	31.99	0.001

*Incomplete or missing item-level missing data on one or more items resulted in the exclusion of the questionnaires from the computation of total score means.*

**Table 7:** Self-reported depressive symptoms of cocaine dependent subjects.

	Pre-Ibogaine	Discharge	1 Month	*F*	*P*
Beck Depression Inventory Total Score Mean	14.3 (3.9) (*n* = 82)	4.2 (1.0) (*n* = 76)	4.5 (1.5) (*n* = 35)	36.86	0.0001
POMS Depression Subscale Total Depression/Dejection Mean	19.4 (15.4) (*n* = 81)	7.1 (6.7) (*n* = 76)	5.8 (5.2) (*n* = 35)	26.21	0.0001
SCL-90-R Depression Subscale Total Depression Mean	1.2 (0.9) (*n* = 81)	0.5 (0.6) (*n* = 81)	0.3 (0.3) (*n* = 32)	29.38	0.0001

*Incomplete or item-level missing data on one or more items resulted in the exclusion of the questionnaires from the computation of total score means.*

Mood scores were significantly improved post ibogaine administration in the cocaine group at all time points (**Table 7**; *p* < 0.0001). Compared to the opioid dependent subjects, the BDI total mean scores showed a more rapid decline and improvement in self-reported depression symptoms at program discharge. Subjects in the cocaine group scored lower on all three assessments at discharge and at 1-month follow up assessments. These results demonstrate that ibogaine administration resulted in a reduction in the severity of depression in acutely abstinent subjects after detoxification from cocaine.

### Self-Reports of Ibogaine Treatment

Oral doses of ibogaine (10–12 mg/kg) were reported to produce a period of active visualizations, beginning approximately 30–45 min after ingestion (**Table 8**). Sensory and perceptual changes included reports of visual images, changes in the quality and rate of thinking, and heightened sensitivity to sound. Most subjects reported a dream-like experience lasting between 4 and 8 h, after which there was an abrupt change in the sensory experience to a more quiet period of deep introspection.

**Table 8:** Self-reports of ibogaine experience.

Connection to higher power, universe	58.3%
Dreamlike state	45.0%
Self as child	43.3%
Able to resist/control experience ^∗^	
Cocaine-dependent subjects	40.0%
Opiate-dependent subjects	16.7%
As film or movie	36.7%
Passive/outside observer	28.3%
Life review	16.7%
Unaware of reality/immersed in experience	11.7%

*Semi-structured elicitation narrative and content coding were used to capture common elements. ^∗^Category with observed differences between opioid and cocaine dependence groups. *N* = 60; Demographics of cocaine and opioid dependent subjects are shown in Supplementary Table S3. ^∗^Category with observed differences between groups.*

We used a narrative elicitation protocol with open-ended questions to gather information about the ibogaine experience in opioid and cocaine dependent subjects (*N* = 60; Supplementary Table S3). Visual images or distortions of vision were reported by 61.7% of subjects. **Table 8** lists common elements obtained from content coding of subjective reports from drug dependent patients treated with ibogaine. Although the reports were varied, many subjects reported that the ibogaine experience was like a “waking dream state” or that they felt that “they were watching a film or a movie” (**Table 8**). Often, there was reported autobiographical content that centered on early childhood experiences (43.3%). Subjects interviewed frequently reported that they had gained an awareness of their connection to their higher power, the universe or a divine presence (58.3%).

Subjects were asked questions to obtain their interpretation of the benefit of the ibogaine experience (**Table 9**). A total of 92% of the subjects reported that they felt a benefit of the experience and that ibogaine was useful as a treatment for drug abuse. Subjects described that they had gained insight into the self-destructive behaviors and that they were mindful of the need to become sober/abstinent now. Some described that they saw images of their death and that they gained an impending awareness of their self-destruction if they failed to become abstinent (18.3%). Many of the most intractable drug abusers reported that they felt “cleansed” or reborn (50%) and that they were given a second chance at life (40%). Only 16.7% of the subjects reported that they would be willing to repeat the ibogaine treatment.

**Table 9:** Frequently reported interpretations of the ibogaine experience.

Useful for drug problems	91.7%
Given insight	86.7%
Need to become sober/abstinent now	68.3%
Cleansed/healed/reborn	50.0%
Second chance at life	40.0%
Increased self-confidence	33.3%
Impending self-destruction if drug use continued	18.3%
Willingness to repeat ibogaine experience	16.7%

*Semi-structured elicitation narrative and content coding. *N* = 60; Demographics of cocaine and opioid dependent subjects are shown in Supplementary Table S3.*

## Discussion

Lotsof (1985) was issued a patent describing “a rapid method for interrupting the narcotic addiction syndrome by administering an oral dose of ibogaine.” His invention claimed, “to provide an improved method for interrupting the physiological and psychological aspects of the heroin addiction syndrome.” The treatment involved oral administration of ibogaine or its salts in dosage ranges of 6–19 mg/kg. The invention claimed a “minimum effective dose to be 400 mg and dosage increases above 1000 mg were found to be unnecessary.” Our observational study of patients seeking opioid detoxification confirms the original claims made by Howard Lotsof. We observed that single oral dose administration of ibogaine was an effective treatment for opioid detoxification. Ibogaine decreased drug craving and improved depressive symptoms when administered in a range of 500–1000 mg. This dosage range appears to be a safe and effective treatment for interrupting the opioid addiction syndrome. Similar benefits were observed in recently abstinent cocaine abusers seeking to interrupt their intractable cycle of drug abuse.

### Safety of Ibogaine

The safety of oral doses of ibogaine was evaluated in a dose range finding study for 191 subjects who elected to undergo detoxification from opioids and cocaine. All clinical laboratory values, observational data, cardiac monitoring, neurologic and psychiatric assessments obtained during the active drug monitoring phase for safety variables were evaluated by licensed physicians. Subjects were self-referred for medically supervised detoxification from opioids (heroin or methadone) or cocaine and met inclusion criteria following a physician’s review of the history and physical examination. The subjects were closely monitored for vital signs for 24 h and side effects up to 7 days after ibogaine administration. The AEs were rated as mild, moderate and severe. All subjects were assessed for nausea and vomiting, headache, ataxia of gait, orthostasis, hallucinations, and other complaints. Clinical laboratory assessments were done immediately prior to administration of the active dose and on the morning after drug administration.

Mild ataxia of gait and nausea and vomiting were seen during the acute drug phase. The period of active oneirophrenic visualization usually resolved within 6–12 h post dose in keeping with the time course for pharmacokinetic clearance of ibogaine. Some subjects reported mild subjective effects at discharge (24 h post-dose), but these remitted shortly thereafter. Ibogaine was well tolerated in the dose range (8–12 mg/kg) in males and females. Respiration rate, systolic and diastolic blood pressures and pulse were unremarkable with only minor changes observed from baseline measures following ibogaine administration. However, bradycardia and hypotension was observed in some cocaine-dependent subjects, which resolved with volume repletion (Mash et al., 1998, 2000). Our study was designed to minimize the risk of possible high dose toxicity and ibogaine-drug interactions in patients which could lead to AEs following ibogaine administration. There were no serious AEs or deaths that occurred from administration of ibogaine to drug dependent patients in the dose range used in this study.

Unfortunately, deaths related to ibogaine have been described for persons seeking detoxification from drugs and alcohol involving variable product purities of ibogaine (HCl or extract) (Alper et al., 2012; Noller et al., 2018). Many of the forensic investigations of ibogaine deaths lacked postmortem toxicologic measures of ibogaine or its metabolite noribogaine in blood (Alper et al., 2012). Ibogaine fatalities are frequently associated with higher doses of ibogaine (>20 mg/kg) which are well above those used in our study, suggesting that there is an increased risk for toxicity at higher doses depending on CYP2D6 genotype. Also, multiple doses of ibogaine “stacked” over time following the initial “flood” dose were reported for many of these cases. The available pharmacokinetic data in humans suggest that administration of multiple doses of ibogaine over time will increase the area under the concentration curve for noribogaine, which would lead to very high levels of ibogaine and the active metabolite noribogaine in blood (Mash, 2018). A review of the available information suggests advanced drug-related comorbidities and contributing conditions, including cardiovascular disease and polydrug abuse in the days or hours prior to ibogaine treatment may have contributed to the AEs and possible drug related fatalities (Kontrimavici et al., 2006; Kubiliene et al., 2008; Alper et al., 2012). Because ibogaine is a medicinal investigational product, these observations underscore the importance of strict inclusion/exclusion criteria to ensure patient safety.

Ibogaine and noribogaine interact with hERG channels *in vitro* (∼5 micromolar; Koenig et al., 2014) and QTc prolongation has been reported in some subjects (Hoelen et al., 2009; Alper et al., 2012). Other opioid agonists approved for clinical use, including methadone, fentanyl, and buprenorphine are effective inhibitors of hERG current, with IC50 values in the 1–10 micromolar range (Katchman et al., 2002). The level of QT prolongation observed at higher plasma levels of ibogaine warrants careful consideration and ECG monitoring. We required cardiovascular assessments to identify persons with pronounced QT prolongation who would be at risk for a possible fatal arrhythmia. Any persons with a bradycardic heart rate below 50 bpm or a long QT syndrome were excluded from our study.

### Opioid Withdrawal Blockade

Lotsof (1985) originally described primary and secondary effects of ibogaine that were time dependent, including an active visualization phase that was followed by a blockade of withdrawal symptoms. He also reported that for some subjects, there was a complete loss of the desire to use heroin for days to weeks after the treatment, suggesting long-lasting after effects of ibogaine. We were the first group to identify 12-hydroxyibogamine (noribogaine) as the active metabolite of ibogaine (Hearn et al., 1995; Mash et al., 1995). We provided evidence that genetic polymorphisms influence the biotransformation of ibogaine in humans, resulting in complex pharmacokinetics (Hearn et al., 1995; Obach et al., 1998; Mash et al., 2000; see also, Glue et al., 2015b). We reported that ibogaine undergoes extensive first pass metabolism in the gut and liver to noribogaine and suggested that this observation may explain the different time course for beneficial after effects of ibogaine administration since ibogaine is cleared from the blood within 14–24 h for most subjects.

Physician ratings of the objective signs of opioid withdrawal demonstrate that ibogaine brings about a rapid detoxification from heroin and methadone. The subjects included in our study identified opioids as one of the primary reasons for seeking ibogaine detoxification. All of the subjects demonstrated active dependence on opioids by clinical evaluation and observations, and confirmed by positive urine screen at program entry. At 36 h post-ibogaine administration, the objective opioid withdrawal score was significantly lower than baseline measures at 2 h prior to ibogaine administration. The average half-life of ibogaine in blood was 1.6–6 h, suggesting that the lasting after effects of the drug were likely due to the CNS activity of noribogaine.

The acute withdrawal syndrome begins approximately 8 h after the last heroin dose, peaks in intensity at 1–2 days with subjective symptoms resolving within 7–10 days. Subjects’ self-reports of withdrawal symptoms 72 h after recovery from ibogaine treatment were significantly decreased from the pre-ibogaine rating and were comparable to the level of discomfort reported at program discharge (Mash et al., 2001). These studies demonstrate that ibogaine effectively blocks the acute signs of opioid withdrawal and the drug cravings and depression associated with the post-acute withdrawal syndrome. Ibogaine treatment brought about a rapid reduction in Becks Depression Index scores (BDI-II) in the New Zealand study (Noller et al., 2018), in agreement with our earlier results that showed clinical improvements in mood and anxiety following oral doses of ibogaine (Mash et al., 1998, 2001).

Ibogaine administration has significant differences among the populations of fast and intermediate metabolizers with regard to maximal concentration, half-life for elimination and the area under the curve of the parent drug and metabolite (Mash et al., 1998). Most of the subjects in this study reported that the active period of dream-like visions would abruptly cease between 6 to 8 h after drug administration. This observation is in keeping with the half-life of ibogaine in blood depending on CYP2D6 genotypes. Poor metabolizers who are CYP2D6 deficient experienced a very pronounced experience that lasted up to 24 h (data not shown; Mash et al., 1998). If the beneficial after effects of ibogaine result from the CNS activity of noribogaine, the CYP2D6 phenotype may be an important determinant in the clinical pharmacology and safety of ibogaine. Pharmacokinetic measurements obtained from human drug-dependent patient volunteers who had received single oral doses of ibogaine (Mash et al., 1998, 2000; Mash, 2018) demonstrate that most of the drug was eliminated (>90%) at 24 h after administration in CYP2D6 fast and intermediate metabolizers. The pharmacokinetic modeling demonstrates that noribogaine remains elevated at 24 h (Mash et al., 1998, 2000, 2001; Glue et al., 2015a). Blood concentration-time effect profiles for single oral doses demonstrate that ibogaine is a pro-drug and noribogaine is a long-acting metabolite.

## Conclusion

Ibogaine reportedly has helped people transition from heroin and cocaine to sobriety (Alper et al., 2008; Brown and Alper, 2017; Davis et al., 2017; Noller et al., 2018). Because of ibogaine’s oneiric effects and complex pharmacokinetics, we originally suggested that noribogaine should be advanced for opioid detoxification to promote a transition to abstinence (Mash et al., 2000, 2001; Mash, 2018). The most effective method currently available for opioid detoxification is substituting and tapering methadone or buprenorphine to treat withdrawal symptoms (Kleber, 2007; Ling et al., 2009; Volkow et al., 2014). Alpha-2 adrenergics (clonidine and lofexidine) can be used to substitute for opioid agonist therapy. Neither of these methods are associated with better long-term outcomes, which mostly appear to be related to post detoxification treatment (Amato et al., 2008).

Detoxification of opioid dependent patients is needed not only to promote a rapid improvement following abrupt cessation of use, but also for sustained effects on diminishing the post-acute withdrawal symptoms that may persist for days to weeks. Ibogaine and its active metabolite noribogaine offer an alternative approach to target the underlying neuroadaptations in the addiction circuits, and which contribute to an intractable cycle of relapse following abstinence. Ibogaine administration unlike a methadone or buprenorphine taper is a rapid detoxification method, shortening the time needed for withdrawal to 2–3 days. The after effects of ibogaine are likely mediated by noribogaine, which may explain the lasting improvement in mood and diminished drug cravings for opioids and cocaine reported by most subjects in this report.

High rates of depressive disorders are reported among people seeking treatment for substance abuse disorders (for review, Nunes and Levin, 2004). The rapid improvement in depressive mood following ibogaine administration may offer an additional benefit for opioid detoxification when compared to an opioid substitution taper or lofexidine as withdrawal agents. Controlled studies are needed to determine effect sizes for ibogaine (or noribogaine) on depressive symptoms that develop during a chronic period of opioid use as compared to a depression that emerges during cessation of use. However, the rapid antidepressant after effects may be an important therapeutic benefit of ibogaine for opioid detoxification.

Ibogaine may help opioid dependent patients to transition to sobriety and to establish a substance-free recovery because the oneirophrenic effects have therapeutic benefit as an adjuvant to psychotherapy (Alper et al., 1999; Davis et al., 2017; Mash, 2018). The elicitation narratives described in this report suggest that ibogaine may promote harm reduction following detoxification from opioids. In addition, lasting after effects of noribogaine may target sites in the addiction circuit in brain to diminish the intractable cravings and desire to use opioids that set into motion the addiction relapse cycle (Baumann et al., 2001; Mash et al., 2016). Claims of high rates of abstinence have been made for ibogaine months after detoxification (Brown and Alper, 2017; Noller et al., 2018), but no verification currently exists and the study cohorts and methods are not comparable. The difficulty in obtaining longitudinal follow up assessments of drug dependent subjects treated outside of the United States remains a major limitation of all published studies to date.

Ibogaine is a psychoactive drug that today is frequently used for detoxification from opioids, even though it has never been licensed as a therapeutic drug. In spite of the lack of therapeutic evidence from well-designed clinical trials, open-label observations in patient volunteers support the conclusion that ibogaine should be considered for clinical development as a medication assisted therapy to help patients transition from opioid maintenance to drug-free abstinence.

Opioid use disorder is a deadly disease that costs the US healthcare system hundreds of billions of dollars each year (Rudd et al., 2016). Controlled clinical trials of ibogaine for opioid detoxification are needed to demonstrate the benefits and risks in human drug review to advance this drug product to market. However, clinical trials take many years and countless millions of dollars to gain FDA approval. Given that traditional approaches to develop new treatments for opioid use disorder have not been advanced by the pharmaceutical industry, ibogaine may be non-addictive alternative that deserves fast-track review as a possible solution to the current opioid drug crisis in America.

## Author Contributions

DM, LD, and KA-F designed the study and collected the data. DM and LD performed the analysis and DM wrote the manuscript. BP developed methodology and contributed to the analysis.

## Conflict of Interest Statement

The author DM is an inventor on issued patents pertaining to the active metabolite of ibogaine. She is a founder and shareholder in a Florida corporation, DemeRx, Inc., which is advancing clinical trials of ibogaine and noribogaine for opioid detoxification and maintenance therapy, among other indications. The author conducted offshore ibogaine research and development studies with government approval in St. Kitts, West Indies. The other authors declare that the research was conducted in the absence of any commercial or financial relationships that could be construed as a potential conflict of interest.
