# Supplementary material for: Ibogaine Detoxification Transitions Opioid and Cocaine Abusers Between Dependence and Abstinence: Clinical Observations and Treatment Outcomes
Source: Front Pharmacol. 2018 Jun 5;9:529. doi: 10.3389/fphar.2018.00529 (PMC5996271; doi:10.3389/fphar.2018.00529)
Supplement: Supplementary file 1 [file Data_Sheet_1.docx]

**Supplemental Table 1. ASI composite scores for opioid dependent subjects**

|  | Pre-Ibogaine  (N= 96) | 1 Month  (N= 33) | 1 year  (N= 39) | F |
| --- | --- | --- | --- | --- |
| Medical | 0.216 (.353) | 0.093 (.249) | - 1. (.271) | 2.01 |
| Employment | 0.390 (.280) | 0.478 (.243) | - 1. (.219) | 1.93 |
| Alcohol Use | 0.130 (.209) | 0.134 (.190) | - 1. (.135) | 0.05 |
| Drug Use | 0.292 (.119) | 0.122 (.123) | - 1. (.101) | 47.79*** |
| Legal | 0.112 (.220) | 0.076 (.162) | - 1. (.143) | 1.99 NS |
| Family/Social | 0.299 (.256) | 0.110 (.135) | 0.206 (.248) | 8.45 ** |
| Psychological | 0.333 (.256) | 0.181 (.201) | - 1. (.226) | 7.89 ** |

All data reflect 30-day information prior to interview. Scores range from 0 to 1

with higher scores indicating greater severity. Mean + SD

* p< 0.05; ** p< 0.001; *** p< 0.0001

**Supplemental Table 2. ASI composite scores for cocaine dependent subjects**

|  | Pre-Ibogaine  (N= 84) | 1 Month  (N= 33) | 1 year  (N= 43) | F |
| --- | --- | --- | --- | --- |
| Medical | 0.146 (.277) | 0.222 (.331) | - 1. (.316) | 0.76 |
| Employment | 0.375 (.287) | 0.353 (.262) | - 1. (.194) | 3.04 * |
| Alcohol Use | 0.215 (.263) | 0.104 (.095) | - 1. (.165) | 4.42 * |
| Drug Use | 0.214 (.101) | 0.104 (.055) | - 1. (.081) | 32.40 *** |
| Legal | 0.132 (.214) | 0.139 (.210) | - 1. (.143) | 2.30 |
| Family/Social | 0.350 (.241) | 0.154 (.207) | - 1. (.262) | 11.21 *** |
| Psychological | 0.347 (.230) | 0.191 (.192) | - 1. (.250) | 7.04 ** |

All data reflect 30-day information prior to interview. Scores range from 0 to 1

with higher scores indicating greater severity. Mean + SD,

* p< 0.05; ** p< 0.001; *** p< 0.0001
