# Supplementary material for: Ibogaine Detoxification Transitions Opioid and Cocaine Abusers Between Dependence and Abstinence: Clinical Observations and Treatment Outcomes
Source: Front Pharmacol. 2018 Jun 5;9:529. doi: 10.3389/fphar.2018.00529 (PMC5996271; doi:10.3389/fphar.2018.00529)
Supplement: Supplementary file 2 [file Data_Sheet_2.docx]

**Supplemental Table 3. Demographic Characteristics of Drug Dependent Participants:**

**Elicitation Narrative (N= 60)**

| Variable | *Total (N=60)* | Opioids (n= 31) | Cocaine (n= 29) |
| --- | --- | --- | --- |
| Gender – Female | 28.3% | 35.5% | 20.7% |
| - Male | 71.7% | 64.5% | 79.3% |
| Age, Mean ± SD | 35.4 ± 8.7 | 33.3 ± 8.5 | 37.6 ± 8.9 |
| Ethnicity , % of subjects |  | | |
| Caucasian | 88.3%  (n= 53) | 96.8%  (n=30) | 79.4%  (n= 23) |
| Hispanic | 6.7%  (n= 4) | 3.2%  (n= 1) | 10.3%  (n= 3) |
| Native American | 5.0%  (n= 3) | 0.0%  (n=0) | 10.3%  (n= 3) |
| Years of Education, Mean ± SD | 14.3 ± 2.2 | 14.6 ± 2.1 | 13.9 ± 2.3 |
| Years of primary drug use, Mean ± SD | 9.5 ± 8.0 | 10.9 ± 8.2 | 12.7 ± 5.0 |
| Number of Previous Drug Treatments, Mean ± SD | 5.8 ± 7.8 | 6.3 ± 11.1 | 4.7 ± 6.0 |

| *Coexisting Axis I – II Disorders* |  |  |  |
| --- | --- | --- | --- |
| Anxiety Disorders except PTSD | 30.0%  (n= 18) | 41.9%  (n= 13) | 17.2%  (n= 5) |
| Bipolar Disorder | 16.7%  (n= 10) | 3.2%  (n= 1) | 31.0%  (n= 9) |
| Depressive Disorders | 48.3%  (n= 29) | 54.8%  (n= 17) | 41.4%  (n= 12) |
| Obsessive-Compulsive Disorder | 0.0%  (n= 0) | 0.0%  (n= 0) | 0.0%  (n= 0) |
| Posttraumatic Stress Disorder | 6.7%  (n= 4) | 12.9%  (n= 4) | 0.0%  (n= 0) |
| Attention-Deficit Disorder (II) | 15.0%  (n= 9) | 3.2%  (n= 1) | 27.6%  (n= 8) |
| Antisocial Personality Disorder (II) | 30.0%  (n= 18) | 35.5%  (n= 11) | 24.1%  (n= 7) |
| Borderline Personality Disorder (II) | 26.7%  (n= 16) | 22.6%  (n= 7) | 31.0%  (n= 9) |
| Schizotypal/Schizophreniform PD (II) | 10.0%  (n= 6) | 12.9%  (n= 4) | 6.9%  (n= 2) |
